# Shallow whole genome sequencing approach to detect Homologous Recombination Deficiency in the PAOLA-1/ENGOT-OV25 phase-III trial

**DOI:** 10.1038/s41388-023-02839-8

**Published:** 2023-11-09

**Authors:** Celine Callens, Manuel Rodrigues, Adrien Briaux, Eleonore Frouin, Alexandre Eeckhoutte, Eric Pujade-Lauraine, Victor Renault, Dominique Stoppa-Lyonnet, Ivan Bieche, Guillaume Bataillon, Lucie Karayan-Tapon, Tristan Rochelle, Florian Heitz, Sabrina Chiara Cecere, Maria Jesús Rubio Pérez, Christoph Grimm, Trine Jakobi Nøttrup, Nicoletta Colombo, Ignace Vergote, Kan Yonemori, Isabelle Ray-Coquard, Marc-Henri Stern, Tatiana Popova

**Affiliations:** 1 Genetics Laboratory, Department of Diagnostic and Theranostic Medicine, Institut Curie and Paris Sciences Lettres Research University, Paris, France; 2 Medical Oncology Department, Institut Curie and Paris Sciences Lettres Research University, Paris, France; 3 Inserm U830, DNA Repair and Uveal Melanoma (D.R.U.M.) Team, Institut Curie and Paris Sciences Lettres Research University, Paris, France; 4 Clinic Bioinformatics Unit, Department of Diagnostic and Theranostic Medicine, Institut Curie and Paris Sciences Lettres Research University, Paris, France; 5 ARCAGY Research, Paris, France; 6 Department of Pathology, University Cancer Institute of Toulouse-Oncopole, Toulouse, France; 7 Biology of Cancer laboratory, University Hospital of Poitiers, Poitiers, France; 8 Department of Gynecology and Gynecologic Oncology, Kliniken Essen-Mitte, Essen, Germany; 9 Department for Gynecology with the Center for Oncologic Surgery Charité Campus Virchow-Klinikum, Charité—Universitätsmedizin Berlin, Corporate Member of Freie Universität Berlin, Humboldt-Universität zu Berlin, and Berlin Institute of Health, Berlin, and AGO, Berlin, Germany; 10 Department of Urology and Gynecology, Istituto Nazionale Tumori IRCCS Fondazione G. Pascale, Napoli, and MITO, Napoli, Italy; 11 Hospital Reina Sofia, Cordoba, and GEICO Group, Córdoba, Spain; 12 Division of General Gynecology and Gynecologic Oncology, Comprehensive Cancer Center, Medical University of Vienna, Vienna, and AGO Austria, Vienna, Austria; 13 Department of Oncology, Copenhagen University Hospital—Rigshospitalet and NSGO, Copenhagen, Denmark; 14 Dipartimento Medicina e Chirurgia, Università Milano-Bicocca, Istituto Europeo Oncologia, Milano, and MaNGO, Milano, Italy; 15 University Hospital Leuven, Leuven Cancer Institute, and BGOG, Leuven, Belgium; 16 Department of Medical Oncology, National Cancer Center Hospital, Tokyo, and GOTIC, Tokyo, Japan; 17 Centre Léon BERARD, and University Claude Bernard Lyon I, Lyon, and GINECO, Lyon, France

**Keywords:** Predictive markers, Genomic instability

## Abstract

The bevacizumab (bev)/olaparib (ola) maintenance regimen was approved for *BRCA1/2*-mutated (BRCAmut) and Homologous Recombination Deficient (HRD) high-grade Advanced Ovarian Cancer (AOC) first line setting, based on a significantly improved progression-free survival (PFS) compared to bev alone in the PAOLA-1/ENGOT-ov25 trial (NCT02477644), where HRD was detected by MyChoice CDx PLUS test. The academic *shallowHRDv2* test was developed based on shallow whole-genome sequencing as an alternative to MyChoice. Analytical and clinical validities of *shallowHRDv2* as compared to MyChoice on 449 PAOLA-1 tumor samples are presented. The overall agreement between *shallowHRDv2* and MyChoice was 94% (369/394). Less non-contributive tests were observed with *shallowHRDv2* (15/449; 3%) than with MyChoice (51/449; 11%). Patients with HRD tumors according to *shallowHRDv2* (including BRCAmut) showed a significantly prolonged PFS with bev+ola versus bev (median PFS: 65.7 versus 20.3 months, hazard ratio (HR): 0.36 [95% CI: 0.24–0.53]). This benefit was significant also for *BRCA1/2* wild-type tumors (40.8 versus 19.5 months, HR: 0.45 [95% CI: 0.26–0.76]). *ShallowHRDv2* is a performant, clinically validated, and cost-effective test for HRD detection.

## Introduction

Homologous recombination deficiency (HRD) associated with inactivation of mainly *BRCA1* or *BRCA2* (*BRCA1/2*) genes plays a major role in tumorigenesis of breast and ovarian cancers, has diagnostic and prognostic value for clinical management of the patients and recently emerged as a biomarker for the treatment targeting repair pathways. *BRCA1/2*-deficient tumor cells impaired for the homologous recombination repair (HRR) rely on alternative pathways to repair DNA double-strand breaks and avoid cell death. However, the use of these alternative repair pathways, including non-homologous end joining, microhomology-mediated end joining and single strand annealing results in numerous and specific alterations accumulated in the cancer genome, which constitute the HRD genomic scars [1–5]. Functioning of all alternative pathways rely on PARP1 and PARP2 enzymes and inhibition of PARP1/2 enzymes in *BRCA1/2*-deficient tumor cells results in cell death [6–8]. PARP inhibitors (PARPi) represent a major progress in the treatment of *BRCA1/2*-deficient tumors. In addition to *BRCA1/2*, only *PALB2* and *RAD51* paralogs inactivation was shown to be consistently associated with tumor HRD in small but noticeable proportion of cases [9]. Even though HRD is largely explained by *BRCA1/2*, *PALB2,* and *RAD51* paralogs, selecting patients by mutation screening would be incomplete due to frequent gene inactivation by other mechanisms (including promoter methylation in *BRCA1* or *RAD51C*) and rare unknown conditions that might accumulate up to 10% of HRD cases [5]. Genomic scar was proven to be a robust biomarker of HRD, which is universal and easy to obtain by the genome sequencing or other custom assays [1–5]. The Myriad MyChoice® CDx Plus test (MyChoice) is a proprietary assay that identifies HRD tumors by combining *BRCA1/2* sequencing with a comprehensive assessment of genomic scars [2–4], providing genomic instability score (GIS). *BRCA1/2*-mutated (BRCAmut) tumors, or *BRCA1/2*-wild-type (BRCAwt) with a GIS ≥ 42, are deemed HRD according to MyChoice, whereas BRCAwt cases with GIS < 42 are considered as nonHRD or HRP (homologous recombination proficient). Sequencing of HRR gene panel (including *ATM*, *BARD1*, *BRCA1/2*, *BRIP1*, *CDK12*, *CHEK1*, *CHEK2*, *FANCL*, *PALB2*, *PPP2R2A*, *RAD51B*, *RAD51C*, *RAD51D*, *RAD54L*) is also performed.

PARPi have been extensively tested in patients with ovarian cancer. Six phase III clinical trials have demonstrated that maintenance with a PARPi, with or without bevacizumab (bev), following platinum-based therapy in patients with newly diagnosed advanced ovarian cancer (AOC) improved progression-free survival (PFS) for BRCAmut and for BRCAwt HRD cases [10–15]. A significant PFS and overall survival (OS) benefit was observed in the PAOLA-1 trial (NCT02477644) with the olaparib (ola) plus bev maintenance regimen compared to bev alone in AOC patients with HRD tumors, where HRD was detected by MyChoice and included BRCAwt cases. It was recently shown in a PAOLA-1 sub-study that BRCAwt HRR gene panels do not predict PFS benefit from maintenance with ola+bev compared with bev, highlighting the importance of HRD genomic scar [16]. Ola+bev maintenance regimen was thus approved in USA, Europe and Japan for patients with BRCAmut or HRD ovarian tumors. However, MyChoice gave a substantial part of non-contributive results (~18% in the PAOLA-1 trial) and was centralized until very recently.

The European HRD ENGOT initiative (EHEI) is a unique collaboration of European academic laboratories aiming to provide new reliable biomarkers for HRD in AOC to select patients who benefit from PARPi ± bev in the first-line maintenance [17]. We have previously developed *shallowHRD*, a genomic scar assay based on low-coverage shallow whole genome sequencing (sWGS) that showed good performance on fresh frozen samples with sensitivity of 87.5% and specificity of 95.2% [18]. sWGS is a quick, reliable and cheap technique, which can be easily applied to clinical samples, including formalin-fixed paraffin embedded (FFPE) samples. The bioinformatics pipeline of *shallowHRD* is straightforward and computationally light with the number of large genomic alterations (LGA, the number of copy number breaks between chromosomal segments at least 10 Mb in size) as a biomarker and two cut-offs (“sensitive” = 15 and “specific” = 20) for HRD largely validated previously [2–4, 18]. However, the approach needed improvements for qualifying as an efficient test for HRD detection in clinical settings. To achieve this goal, we developed *shallowHRDv2* that (i) secures correct estimation of LGA by managing specific noise coming from FFPE samples and (ii) minimizes not conclusive diagnostics by resolving the cases between “sensitive” and “specific” cut-offs. Clinical validation of *shallowHRDv2* on PAOLA-1 and routine samples demonstrated excellent performance, paving the path for clinical application.

## Results

Institut Curie joined the EHEI initiative and had access to 449 DNA extracted from FFPE tumor samples from the PAOLA-1 trial to validate the *shallowHRDv2* test. Characteristics of these 449 patients at baseline were representative of the global cohort (Supplementary Table 1). All samples were selected because of the availability of the material and were previously tested by MyChoice in the frame of the PAOLA-1 trial; Institut Curie did not have access to the HRD/BRCAmut tumor status until the *shallowHRDv2* test was performed.

**Fig. 1:**
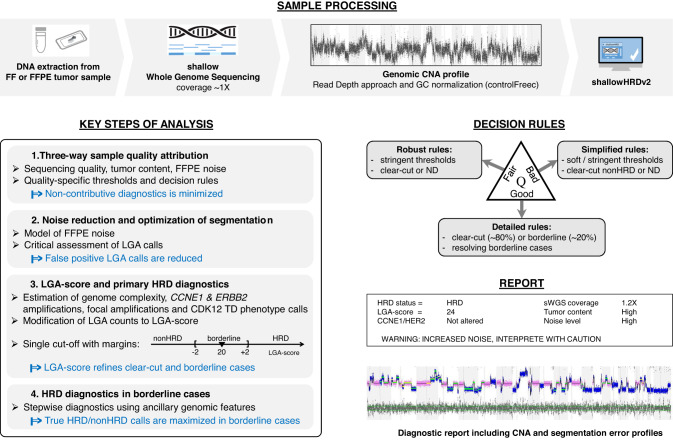
Shallow WGS approach and the main steps of shallowHRDv2 test for effective HRD diagnostics. CNA Copy number alteration, FF Fresh frozen, FFPE formaldehyde fixed and Paraffin embedded, HRD Homologous recombination Defect, ND non-determined, TD tandem duplications, LGA large genomic alterations, Q quality.

*ShallowHRDv2* is a software tool trained on pan-cancer series of ~1000 sWGS partially annotated with *BRCA1/2* status that takes as an input normalized copy number alteration (CNA) profile from sWGS (or WGS) and provides HRD status based on LGA-score (Fig. 1). LGA-score essentially corresponds to the number of large genomic alterations [18] modulated by penalties (subtracted if the genomic features known to be associated with nonHRD, such as *CCNE1* or *ERBB2* amplification, were detected) and/or bonuses (added mainly if the CNA pattern is consistent with near-diploid tumor genomic content). *ShallowHRDv2* classification strategy consists in stepwise diagnostics starting from confident nonHRD or HRD status attributed to the “clear-cut” cases (which LGA-scores are beyond the margins around the cut-off) followed by resolving the “borderline” cases (which LGA-scores are within the margins around the cut-off) (see Methods section). In the PAOLA-1 cohort, clear-cut confident diagnostics was applicable to 82% (373/449) of cases, where 57% (211/373) were clear-cut HRD. Among the borderline cases 33% (20/61) were further classified as HRD. Fifteen non-contributive cases were mainly explained by extremely low tumor content or combination of poor-quality DNA with low tumor content and “borderline” scores.

For the 394 samples with conclusive results by both MyChoice and *shallowHRDv2* tests (394/449; 88%), an overall concordance was 94% (369/394) with positive agreement of 95% (196/206) and negative agreement of 92% (173/188; Table 1). Cohen’s Kappa at 0.73 (*p* = 1.95 × 10^−148^) indicated a substantial agreement. The percentage of inconclusive tests for MyChoice was 11% (51/449), while *shallowHRDv2* yielded a failure rate of 3% (15/449). The correlation between scores from MyChoice (GIS) and *shallowHRDv2* (LGA-score) was strong (R^2^ = 0.85) and discordant cases were concentrated around the thresholds of each test (Supplementary Fig. 1). Among the 15 nonHRD^GIS^ / HRD^LGA-score^ cases, 9 tumors carried a pathogenic variant in the screened HRR genes: *BRCA1/2* (6 cases), *CDK12* (2 cases) and *RAD51D* (1 case). For the six remaining cases, MyChoice did not report any alteration in the screened HRR genes. Among 10 HRD^GIS^ / nonHRD^LGA-score^ cases, three tumors carried *BRCA1/2* pathogenic variant, two tumors had *CDK12* pathogenic variant and 5 did not have any alteration in the screened HRR genes. The information about a corresponding loss of heterozygosity was not available for *BRCA1/2* and other reported gene variants.

**Table 1:** Concordance between GIS and shallowHRDv2 test on 449 tumor samples from PAOLA-1 patients.

	ShallowHRDv2		
	Positive	Negative	NC
GIS			
Positive	196	10	1
Negative	15	173	3
NC	17	23	11

*NC* non-contributive.

**Fig. 2:**
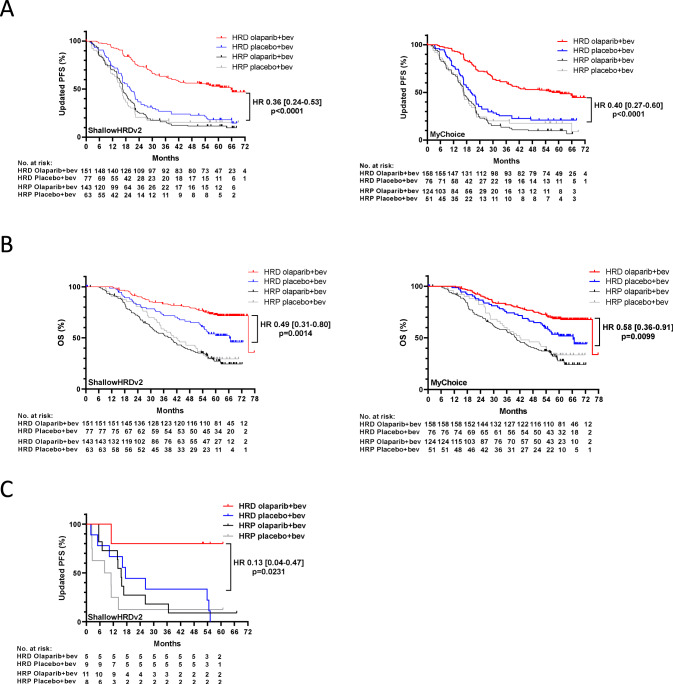
Kaplan–Meier Estimates of Progression-free Survival and Overall survival according to ShallowHRDv2 or Mychoicetest in PAOLA-1 trial. Kaplan–Meier estimates of (**A**) PFS for PAOLA-1 patients according to homologous recombination status as determined by shallowHRDv2 or MyChoice and to the treatment arm. **B** OS for PAOLA-1 patients according to homologous recombination status as determined by shallowHRDv2 or MyChoice and to the treatment arm. **C** PFS according to homologous recombination status as determined by shallowHRDv2 and to the treatment arm for PAOLA-1 patients with non-contributive results by MyChoice. HR Hazard Ratio, PFS Progression Free Survival, OS Overall Survival.

Given the strong overall agreement between *shallowHRDv2* and MyChoice, a similar prediction of clinical benefit of ola + bev maintenance is expected in this subset of the PAOLA-1 cohort [11, 19]. The median duration of follow up was 63 months (interquartile range [IQR]: 28.5–62.3). Median PFS was 65.7 months in the HRD group according to *shallowHRDv2* (whatever the *BRCA1/2* status) treated with ola+bev and 20.3 months when treated with bev (HR 0.36 [95% CI: 0.24–0.53]; *p* < 0.0001), whereas it was 57.1 months in the HRD ola+bev group and 20.1 months in the HRD bev group according to MyChoice (HR 0.40 [95% CI: 0.27–0.60]; *p* < 0.0001 Fig. 2A). Median OS was 75.2 months in the HRD group treated with ola+bev and 66.4 months when treated with bev (HR 0.49 [95% CI: 0.31–0.80]; *p* = 0.0014) and HR 0.58 [95% CI: 0.36–0.91]; *p* = 0.0099) for *shallowHRDv2* and MyChoice, respectively; Fig. 2B).

Importantly, *shallowHRDv2* was also predictive of the PARPi benefit for patients with a non-contributive MyChoice test. Median PFS in HRD patients was not reached when treated with ola+bev as compared with a median PFS of 17.4 months when treated with bev alone (HR: 0.13 [95% CI: 0.04–0.47]; *p* = 0.0231 Fig. 2C).

**Fig. 3:**
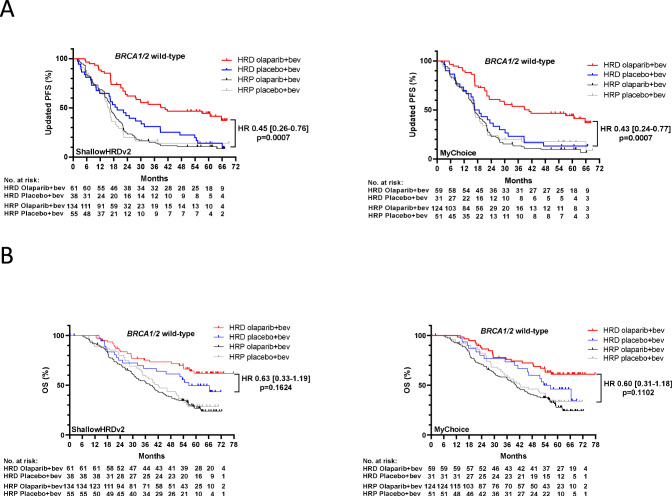
Kaplan–Meier Estimates of Progression-free Survival and Overall survival according to ShallowHRDv2 or Mychoice test in BRCA1/2 wild-type tumors. Kaplan–Meier estimates of (**A**) PFS; (**B**) OS according to homologous recombination status as determined with shallowHRDv2 or MyChoice and to the treatment arm for PAOLA-1 patients with tumors wild-type for *BRCA1/2* genes. HR Hazard Ratio, PFS Progression Free Survival, OS Overall Survival.

The main advantage of detecting HRD by genomic scars is to identify tumors deficient for HRR pathway in the absence of *BRCA1/2* pathogenic variants. Indeed, the predictive values of *shallowHRDv2* and MyChoice in the subgroup of patients with BRCAwt AOC were significant and nearly identical. Median PFS was 40.8 months with ola+bev and 19.5 months with bev for *shallowHRDv2* (HR: 0.45 [95% CI: 0.26–0.76]; *p* = 0.0007) and median PFS was 40.8 months with ola+bev and 17.6 months with bev for MyChoice (HR: 0.43 [95% CI: 0.24–0.77]; *p* = 0.0007) in the subgroup of BRCAwt HRD predicted tumors (Fig. 3A).

Of note, patients with BRCAwt tumors receiving bev alone and classified as HRD by *shallowHRDv2* tended to have a longer PFS than patients classified as HRP (nonHRD) suggesting a prognostic value of the HRD status. However, this difference was not statistically significant (*p* = 0.28). Importantly, this benefit for HRD BRCAwt in PFS was also observed for OS. In HRD BRCAwt tumors, the median OS was not reached in the ola+bev treated patients, whatever the test employed, versus a median OS in bev-treated patients of 56.6 and 55.0 months when HRD was defined by *shallowHRDv2* or MyChoice, respectively (HR for comparison between ola+bev and bev: 0.63 [95% CI: 0.33–1.19]; *p* = 0.1624 and 0.60 [95% CI: 0.31–1.18]; *p* = 0.1102, defined by *shallowHRDv2* or MyChoice, respectively; Fig. 3B). In contrast, patients with HRP BRCAwt tumors according to *shallowHRDv2* receiving ola + bev tended to have a shorter median OS than those receiving placebo+bev although not significant (38.2 versus 42.1 months, respectively; HR: 1.12 [95% CI: 0.78–1.61]; *p* = 0.55, Fig. 3B).

Noteworthy, *shallowHRDv2* displayed significant improvement compared to *shallowHRD* in the same PAOLA-1 subset, delivering diagnostics for 15% (66/449) not conclusive borderline cases, thus identifying 34 HRD patients who must receive PARPi (Supplementary Table 2). Thirty of these 34 cases are also classified as HRD with MyChoice so these 30 patients certainly benefit of a PARPi treatment whereas the clinician cannot prescribe a PARPi treatment with a borderline result. Correct attribution was confirmed by significant PFS difference between HRD and nonHRD groups of patients when receiving ola+bev treatment (HR 0.25 [95% CI: 0.13–0.49]; *p* < 0.0001) (Supplementary Fig. 2). Otherwise, the performances of *shallowHRD* in comparison with MyChoice were acceptable with an overall agreement of 93% (309/333), a positive agreement of 95% (169/177), a negative agreement of 90% (140/156) and 4% (17/449) of non-contributive (besides borderline) cases (Supplementary Table 3). Moderate agreement of 0.56 (*p* = 8.25 × 10^−72^) by Cohen’s Kappa confirms *shallowHRDv2* superiority.

Good analytical performance of *shallowHRDv2* and its equivalency to MyChoice to predict PARPi benefit in the PAOLA-1 cohort were also confirmed in the independent cohort of unselected, consecutive cases from routine clinical diagnosis practice. FFPE AOC cases issued from Institut Curie routine laboratory (109 cases) were processed by *shallowHRDv2* and MyChoice. The high overall agreement of 91% (86/94) with 92% (36/39) positive agreement and 91% (50/55) negative agreement between *shallowHRDv2* and MyChoice, Cohen’s Kappa at 0.69 (*p* = 7.49 × 10^−29^), and less non-contributive results for *shallowHRDv2* (5% versus 12%) confirmed the results of PAOLA-1 clinical trial in the prospective cohort (Table 2).

**Table 2:** Concordance between GIS and shallowHRDv2 test on 109 tumor samples from Institut Curie routine laboratory.

	ShallowHRDv2		
	positive	Negative	NC
GIS			
Positive	36	3	0
Negative	5	50	2
NC	1	8	4

*NC* non-contributive.

The *shallowHRDv2* test has been also validated in a small cohort of patients where sWGS was performed in the University Hospital of Poitiers on 31 DNA samples of FFPE AOC cases issued from their routine laboratory and analyzed by MyChoice. An overall agreement of 90% (26/29) was observed, with 80% (8/10) positive agreement and 95% (18/19) negative agreement. There was 6% of non-contributive analyses (Table 3). High agreement between *shallowHRDv2* and MyChoice on the sWGS obtained outside of Institut Curie confirmed that the *shallowHRDv2* test can be decentralized.

**Table 3:** Concordance between GIS and shallowHRDv2 test on 31 tumor samples from Poitiers Hospital routine laboratory.

	ShallowHRDv2		
	Positive	Negative	NC
GIS			
Positive	8	2	0
Negative	1	18	0
NC	0	0	2

*NC* non-contributive.

## Discussion

Assessment of HRD status is mandatory to balance benefit and risks of PARPi maintenance in newly diagnosed AOC patients. We report here clinical validation of *shallowHRDv2* test in clinical trial and in prospective cohorts. *shallowHRDv2* showed high concordance with MyChoice test in the PAOLA-1 trial to predict the benefit of ola+bev in AOC patients. *shallowHRDv2* improved significantly the performance of *shallowHRD* test but maintained its computational efficiency and overall approach to mine large-scale CNA for HRD recognition [18]. Key factors that boost *shallowHRDv2* performance include FFPE noise reduction, critical assessment of diagnostics based on tumor content and sWGS noise level, and calling ancillary genomic features to refine the conclusion for the borderline scores. *shallowHRDv2* is shown to reduce the number of non-conclusive results by ~60–75% as compared with MyChoice (3% versus 11% in the PAOLA-1 cohort and 5% versus 12% in the routine cohort). More importantly, the correct attribution of the patients with *shallowHRDv2* HRD status non-contributive in MyChoice was confirmed by their significant benefit from ola+bev combination treatment, which potentially allows more patients with AOC to benefit from ola+bev. With only 3.3% of non-contributive test results, s*hallowHRDv2* robustness is similar to other genetic testing in clinical routine, such as *BRCA1/2* tumor mutation testing, which showed a 4.4% failure rate in the PAOLA-1 clinical trial [20].

Analysis of the discordant cases between *shallowHRDv2* and MyChoice (25/449, 5.5%) did not show a systematic bias and there was no ground truth for HRD status available for an objective comparison. Nevertheless, when considering the 10 discordant cases with pathogenic variants in *BRCA1/2*, *RAD51* paralogs, or *PALB2*, which are supposed to be HRD, seven cases were correctly classified as HRD by *shallowHRDv2* and not by MyChoice, while the three others were correctly classified by MyChoice and not by *shallowHRDv2*. Of note, 4 *CDK12* mutated cases were among the discordant ones, probably due to their complex genomic scars, while their sensitivity to PARPi is still questionable [21]. In general, validation of HRD positive but not *BRCA1/2*, *RAD51* paralogs or *PALB2* inactivated cases is limited by the lack of a gold standard for the biological assessment of the true HRD status. Despite their limitations, both tests remain highly effective in identifying patients who benefit from PARPi treatment in the absence of *BRCA1/2* tumor mutation, as observed by PFS analyses. Overall, the analytical and clinical validation presented here showed that the decentralized academic *shallowHRDv2* test is equivalent to MyChoice to identify AOC patients who will benefit of PARPi treatment.

The clinical value of HRD testing may not be restricted to the question of ola in AOC. Contrary to ola, which has not been authorized in first line for HRP cases (alone or in combination with bev), niraparib has obtained an “all-comers” approval. However, the subgroup analysis of the PRIMA trial showed a smaller magnitude of benefit with niraparib in the HRP subgroup, as determined by MyChoice (HR: 0.68 versus 0.40 in HRP and HRD, respectively, with median PFS of 8.1 and 5.4 months with or without niraparib in the HRP subgroup, respectively), limiting the clinical benefit for patients in this situation [19]. Using a cheap and robust HRD test comparable with MyChoice such as *shallowHRDv2* might thus help estimating the benefit of prescribing niraparib in BRCAwt AOC patients in first-line. Similarly, and more globally, HRD testing might be useful for other tumor types besides ovarian carcinomas [22, 23], including prediction of the response to PARPi in other tumor types as already tested in breast cancer [24]. However, *ShallowHRDv2* should not be used in other tumor types before validation in specific sample sets.

Other teams also participated in the EHEI and the new tests detecting HRD have been validated on tumor samples from the PAOLA-1 trial [25–29]. All of them reported a decrease in non-contributory results compared with MyChoice and overall satisfactory clinical performances in predicting PFS benefit from ola+bev combination. However, the majority of these tests are based on NGS capture panels coupled with sequencing of HRR genes. Thus, laboratories implementing these tests would be forced to change their already validated method for HRR gene screening. This is not required for *shallowHRDv2* which is based on pre-capture library sequencing. *ShallowHRDv2* test is very easy to implement in all laboratories with NGS resource. In addition, the strategy of pre-capture library allows performing target sequencing of HRR genes in parallel to sWGS. Single nucleotide polymorphism arrays approach for HRD detection is also independent of HRR genes sequencing but is more expensive and time-consuming compared to sWGS. Considering the reduced technical time and cost of reagents and consumables due to the sequencing of the pre-capture library, we estimate that the *shallowHRDv2* test is around four times less expensive than other available academic or commercial HRD tests, which are billed at between €1500 and €2200 in France in 2023.

In conclusion, academic decentralized *shallowHRDv2* test is robust, cost-effective, easy to implement, clinically validated and can be considered as a reference test to detect HRD, along with the MyChoice test to identify AOC patients who will benefit of PARPi treatment.

## Methods

### Patients and tumor samples

The first cohort consisted of FFPE-derived DNA from 449 AOC samples from the PAOLA-1/ENGOT-ov25 trial in the frame of the EHEI. The procedure consisted in preliminary evaluation of the academic test performance on 85 PAOLA-1 BRCAwt tumors by comparing to the Myriad MyChoice diagnostics, followed by final PFS based test evaluation on 364 additional patient samples (phase 3) [17]. The PAOLA-1 trial was designed by the European network for gynecological oncological trial (ENGOT) lead group, *Groupe d’Investigateurs Nationaux pour l’Etude des Cancers Ovariens* (GINECO), and sponsored by *Association de Recherche sur les Cancers GYnécologiques* (ARCAGY research). This trial was performed in accordance with the provisions of Helsinki declaration and good clinical practice guidelines under the auspices of an independent data monitoring committee. All patients provided written informed consent.

The second cohort consisted of 109 consecutive FFPE AOC samples (8 to 20 slides of 5 µm according to the tumor area) sent to Myriad Genetics central laboratory (Salt Lake City, UT, USA), from March 2021 to January 2022, as part of the routine clinical management process. All patients have a recent diagnosis of high grade AOC and the samples contained more than 30% of tumor cells. *ShallowHRDv2* was performed on DNAs from the same FFPE samples, extracted and sequenced in the Institut Curie genetics laboratory.

The third cohort consisted of 31 consecutive FFPE AOC samples issued from the routine laboratory of Poitiers Hospital previously analyzed by MyChoice test in Myriad Genetics central laboratory. *ShallowHRDv2* was performed in Institut Curie on FASTQ files from sWGS performed in Poitiers Hospital.

### Shallow WGS workflow

Genomic DNA was extracted from the FFPE samples. 50 µl of DNA samples were mechanically sheared using Covaris ME220 Focused-ultrasonicator. Libraries were prepared from 100 ng of DNA using Agilent SureSelect XT HS and XT Low Input Library Preparation kit (Agilent, Santa Clara, CA, USA, ref: G9703A) according to the manufacturer’s instructions. This process included ligation, PCR amplification, and purification on AMpure XP beads (Beckman Coulter, Indianapolis, IN, USA, ref: A63882). DNA concentration was measured using either Thermo Fisher Scientific Qubit® dsDNA HS Assay Kit (ref: Q32854) or Qubit® dsDNA BR Assay Kit (Thermo Fisher Scientific, Waltham, MA, USA, ref: Q32853). Library quality and quantity were assessed using the Agilent TapeStation and D1000 ScreenTape. A pre-capture library pool with concentrations of 4 nM or 1.8 nM was generated for NextSeq 550 S or NovaSeq 6000 Sequencing systems (Illumina Inc, San Diego, CA, USA), respectively.

In Poitiers Hospital, DNA from FFPE samples were extracted on a Maxwell® 16-IVD using FFPE Plus LEV DNA Purification Kit (Promega, Madison, WI, USA, ref: AS1135). 200 ng of FFPE DNA were used for generating pre-capture libraries on a Magnis NGS Prep System using the SureSelect XT HS Low Input kit (ref: G9731D). 100 µl of molecular biology grade water were added to the QC strip wells (blue strip) and quantification was performed with on a Qubit® 3 fluorometer using the 1x dsDNA HS Assay Kit (ref: Q33230). The pre-capture library pool was sequenced at 1 nM on a NextSeq 550 platform (Illumina). After sequencing, FASTQ files were generated and analyzed by *shallowHRDv2*.

### ShallowHRDv2 bioinformatics pipeline

After DNA extraction and whole genome sequencing at low coverage (~1X), read counts profile (bin size ~50 kb) normalized and corrected for GC-content was obtained by ControlFreec [30] (Fig. 1). ShallowHRDv2 bioinformatics pipeline consists in the analysis of copy number alteration (CNA) profile providing HRD diagnostics, sample quality attribution and comprehensive quantitative and graphical output for manual control. Main steps of the pipeline, outline of the decision rules and diagnostics are described below, with more details provided in Supplementary methods. The raw data from sWGS have been deposited at ENA under accession PRJEB61549.

Main steps of CNA processing (Supplementary Fig. 3)*:*

1. Three-way sample quality attribution: CNA profile classification according to tumor content (four categories), intrinsic sWGS noise (three categories) and FFPE noise (four categories) with final integrative classification in three categories: “good”, “fair” and “low” (Supplementary Fig. 4).
2. Noise reduction and optimization of breakpoints in the CNA profile: filtering small segments and assembling segments with small differences or local correlations to the FFPE noise profile (obtained from ~100 normal profiles from FFPE samples, Supplementary Fig. 5), with the thresholds for the breakpoint calls fitted for each quality category.
3. Broad CNA profile characterization by:
  genome complexity, where the “simple” genome has two most abundant copy number (CN) levels accounting for more than 70% of the genome, otherwise, the genome is classified as “complex” or “complex+” (significant and almost equal contribution of four CN levels);
  a set of binary attributes, such as *CCNE1* amplification, *ERBB2* amplification, focal amplification phenotype (called when more than two chromosome arms carry at least one amplification), *CDK12* mutation-associated tandem duplication phenotype (called when multiple interstitial gains of 1–10 Mb are detected) [21];
  a set of parameters characterizing the breakpoints, including the number of large genomic alterations (LGA), that largely contributes to the HRD diagnostics; LGA are defined as CN breaks between genomic segments of more than 9 Mb (segment sizes are rounded to the integer value); a set of ancillary LGA indexes, such as extra-large LGA, telomeric LGA, LGA between two the most abundant CN levels, LGA involving one of the three most abundant CN levels and the number of chromosome arms with LGA.
4. Step-wise HRD diagnostics based on:
  LGA-score, which is essentially the number of LGA modified by PENALTY and/or BONUS, where PENALTY is defined by binary attributes and is subtracted from the LGA number (PENALTY is set to 0, 5 or 8 if none, one or more than one binary attributes hold true, respectively) and BONUS is defined by the genome complexity and is added to the LGA number (BONUS is set to 5 for “simple” genome and to 0 otherwise);
  the threshold of 20 with margins (±2) for clear-cut HRD diagnostics, namely 18 and 22, with the LGA-score <18 for “clear-cut” nonHRD and LGA-score >22 for “clear-cut” HRD;
  LGA-score modification, which is applied to resolve the borderline cases (18 ≤ LGA-score ≤ 22). Briefly, the LGA-score is shifted to 19 if evidence for nonHRD (such as PENALTY > 0, ancillary LGA indexes below the threshold, genome is classified as “complex+”, etc) and to 21 if evidence for HRD (PENALTY = 0, ancillary LGA indexes above the threshold, genome is classified as “simple”) (Supplementary Fig. 6).

#### Decision rules

Decision rules are multi-step, depend on sample quality attribution and include selection of the thresholds for LGA call. Two thresholds were utilized to call LGA at CNA breakpoint: stringent (implying simple genome) and soft (implying complex genome), which are applied in conservative manner for good quality cases (LGA-score was based on LGA number with soft/stringent thresholds for nonHRD/HRD clear-cut diagnostics), and are fixed to the stringent/soft ones for noisy/low tumor content samples, respectively. Simplified decision rules for bad quality samples consist in giving the diagnosis only for clear-cut nonHRD cases with small overall number of the breakpoints. Robust decision rules for fair quality samples consist in giving the diagnosis only for clear-cut cases, leaving the borderline cases with not determined (ND) diagnosis.

#### Comprehensive output (report)

Final diagnosis is reported along with quality assessment and warning messages (Supplementary Fig. 7). Quantitative output provides complete information on the decisive genomic biomarkers, LGA, LGA-score and HRD diagnostics. Output includes segmented profile with the LGAs detected and the error profile to visually control noise reduction quality and segmentation.

Circular binary segmentation of CNA profile has a stochastic component and can result in between-run variation in LGA-score, which may affect the diagnostics if close to the thresholds. Thus, LGA number is represented by the confidence intervals estimated from 20 segmentation/optimization runs.

### Statistical analysis

The Kaplan–Meier method was used to estimate the PFS and overall survival (OS), with the stratified log-rank test used to assess the difference between the ola+bev and bev arms [11, 19]. The hazard ratio (HR) and associated 95% confidence interval (95% CI) were calculated with the use of a stratified Cox proportional-hazards model. The comparison among the different tests for agreement in the detection of HRD was determined using the Cohen’s kappa coefficient. All statistical analyses were performed using GraphPad Prism software version 9.1.0.

### Supplementary information

Supplementary Information

Supplementary Table 1

Supplementary Table 2

Supplementary Table 3

## Data Availability

The raw data from sWGS have been deposited at ENA under accession PRJEB61549.
