# Supplementary material for: Shallow whole genome sequencing approach to detect Homologous Recombination Deficiency in the PAOLA-1/ENGOT-OV25 phase-III trial
Source: Oncogene. 2023 Nov 9;42(48):3556–63. doi: 10.1038/s41388-023-02839-8 (PMC10673712; doi:10.1038/s41388-023-02839-8)
Supplement: Supplementary file 1 — Supplementary Information [file 41388_2023_2839_MOESM1_ESM.pdf]

SUPPLEMENTARY METHODS for

Shallow whole genome sequencing to detect Homologous Recombination Deficiency in the PAOLA-1/ENGOT-OV25 phase-III trial

Celine Callens[1], Manuel Rodrigues[2,3], Adrien Briaux[1], Eleonore Frouin[4], Alexandre Eeckhoutte[3], Eric Pujade-Lauraine[5], Victor Renault[4], Dominique Stoppa-Lyonnet[1], Ivan Bieche[1], Guillaume Bataillon[6], Lucie Karayan-tapon[7], Tristan Rochelle[7], Florian Heitz[8], Sabrina Chiara Cecere[9], Maria Jesús Rubio Pérez[10], Regina Berger[11], Trine Jakobi Nøttrup[12], Nicoletta Colombo[13], Ignace Vergote[14], Isabelle Ray-Coquard[15], Marc-Henri Stern[1,3] and Tatiana Popova[3]

Conceptual workflow of shallowHRDv2

ShallowHRDv2 aims at high confidence and efficiency of HRD prediction based on Copy Number Alteration (CNA) profile obtained from shallow Whole Genome Sequencing (sWGS) of FF (fresh frozen) or FFPE (formalin-fixed paraffin embedded) tumor sample. To achieve this goal, we developed the workflow which (i) secures correct estimation of LGA (large genomic alterations) by the system of adaptive thresholds, quality grading and noise reduction and (ii) minimizes not conclusive diagnostics by resolving the area of uncertainty around the cut-offs for LGA, which were obtained and largely validated previously {Popova et.al 2012, Eeckhoutte et al, 2020}. The conceptual workflow is shown in Supplementary Figure 3 and detailed below.

(1) CNA profile segmentation using the circular binary segmentation method and CNA profile classification according to tumor content (four categories), intrinsic sWGS noise (three categories) and FFPE noise (four categories). Combination of these attributes provides overall sample quality attribute (“good”, “fair” or “bad”), utilized in selection of the decisive pipeline and reporting (Supplementary Figure 4).

- Profile is characterized by the number of breakpoints; variance of CNA profile, within segment variance, between segments variance; correlation to FFPE noise; percent of the genome belonging to the segments >20Mb after segmentation.
- *Sequencing quality* is characterized by raw variance (variance within the segments).

- *Tumor content* is characterized by the variance of medians of the large segments.
- *FFPE noise* is characterized by the number of breakpoints, correlation to FFPE cumulative profile, variance of error profile and the proportion of large segments after initial profile segmentation. FFPE noise profile is obtained from segmented CNA profiles of ~100 almost normal genomes sequenced from FFPE samples. Each segment average is replaced by 1 or -1, if upper or lower than general average value, respectively. Each genomic bin is thus characterized by the sum of 1/0/-1 from ~100 profiles (Supplementary Figure 5). Correlation > 0.2 between tumor CNA and FFPE cumulative profiles and proportion of the large segments after segmentation < 40% are characteristic of high FFPE noise.

(2) Noise correction and segmentation optimization: filtering small segments and merging segments with small difference in median values or local correlations to the FFPE noise profile. A threshold for between segment difference to be considered as negligible (no breakpoint call) is selected depending on the noise and tumor content category. The adjacent segments were merged if the median difference is less than the threshold. The breakpoint(s) were also eliminated if it (they) followed the breakpoint in FFPE covariate profile even if the difference exceeded the threshold.

(3) Genomic profile characterization:

- Estimation of the genome complexity (three categories: “simple”, “complex” and “complex+”), where “simple” genome has two most abundant copy number levels accounting for more than 70% of the genome. Otherwise, the genome is classified as “complex”. “Complex+” is a subtype of “complex” that accounts for more than three equally abundant copy number levels; all low tumor content cases are annotated as “simple”.
- Overall profile quality attribution (three categories: “good”, “fair” and “low”), which is based on tumor content and noise, and defines the path to final diagnosis, including conditions for not determined status (ND).

- CNA breakpoints analysis: LGA calling is performed after filtering out the segments less than 3Mb and merging adjacent large segments if between segment distance is less than 3Mb; LGA are called in adaptive mode, i.e. using two thresholds, stringent (implying simple genome) and soft (implying complex genome); stringent threshold is also applied in the noisy samples, while soft threshold is applied in low tumor content cases.
- detection of *CDK12* mutation-associated (CDK12mut) tandem duplication phenotype based on the number of interstitial gains of 1-10Mb {Popova et al, 2016};
- Check for *CCNE1* amplification, *ERBB2 (*HER2*)* amplification and amplification phenotype (called when more than two chromosome arms carry at least one amplification).

(4) LGA-score and HRD status attribution (Supplementary Figure 6A):

- LGA-score = LGA + BONUS - PENALTY,

  where BONUS=5 for "simple" genome; PENALTY=5 if one amplification or CDK12mut phenotype is detected; PENALTY=8 if two or more of these features are detected.
- LGA-score distribution in the training set is shown in Supplementary Figure 6B. The main threshold for LGA-score in HRD attribution is 20 with margins (±2).The LGA-score less than 18 and the LGA-score more than 22 are considered definitive (clear-cut); the LGA-score more than 17 and less than 23 is considered borderline.
- For borderline LGA-score several modification rules are applied: the LGA-score is shifted to 19 if evidence for nonHRD (for example, PENALTY>0), or the LGA-score is shifted to 21 if evidence for HRD (for example, BONUS>0).
- Ancillary cumulative LGA index, LGA-boost, is used to further clarify the HRD status of borderline cases. LGA-boost is defined as a sum of sub-types of LGAs detected in a tumor genome:

  LGA-boost = LGA_chr_arm + LGA_at_telomere + LGA_20Mb + LGA_baseline + LGA_baseline_12

where LGA_chr_arm is the number of chromosome arms with LGA; LGA_at_telomere is the number of chromosome arms with LGA at telomeric end; LGA_20Mb is the number of LGA with both genomic segments at CN break being more than 19Mb; LGA_baseline is the number of LGA involving the most abundant CN layer; LGA_baseline_12 is the number of LGA detected between two most abundant CN layers.

- HRD is called if LGA-score >20 and nonHRD is called if LGA-score <20. Borderline scores in FAIR or LOW quality samples lead to ND diagnostics.
- Final diagnosis is reported along with quality assessment and warning messages (Supplementary Figure 7).

Random initiation of segmentation algorithm and stochastic profile optimization by the system of fixed thresholds lead to possible variation in LGA number eventually affecting the final diagnosis. The complete workflow thus includes 10 runs to fix intermediate parameters plus 10 runs to get error estimation for LGA counts.

References

Popova, T., et al. Ploidy and large-scale genomic instability consistently identify basal-like breast carcinomas with BRCA1/2 inactivation. Cancer Res 72, 5454-5462 (2012).

Eeckhoutte, A., et al. ShallowHRD: detection of homologous recombination deficiency from shallow whole genome sequencing. Bioinformatics 36, 3888-3889 (2020).

Popova, T., et al. Ovarian Cancers Harboring Inactivating Mutations in CDK12 Display a Distinct Genomic Instability Pattern Characterized by Large Tandem Duplications. Cancer Res 76, 1882-1891 (2016).

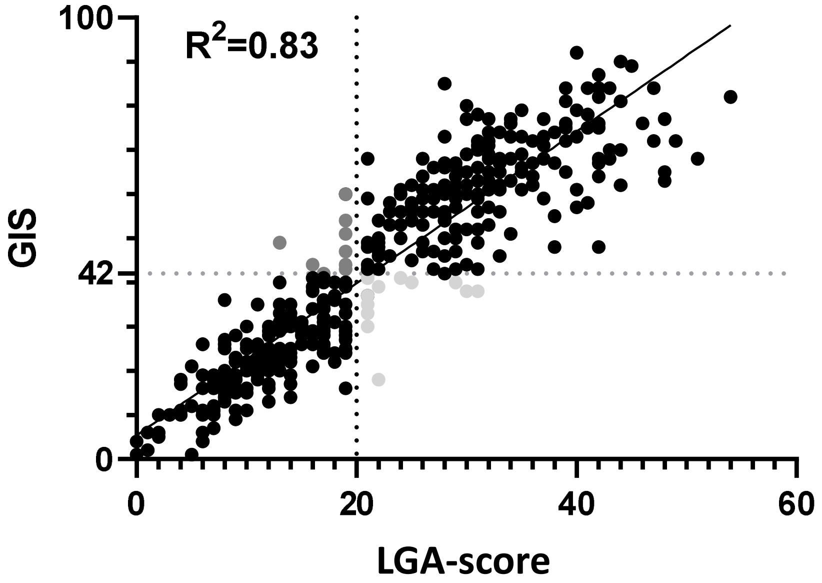

Supplementary Figure 1. Correlation between GIS and LGA-scores. Discordant cases are plotted in grey (HRD^{GIS}/$nonHRD^{LGA\text{-}score}$) and light grey ($nonHRD^{GIS}$/$HRD^{LGA\text{-}score}$). GIS: Genomic instability Score by MyChoice; LGA-score: Large Genomic Alterations score by shallowHRDv2.

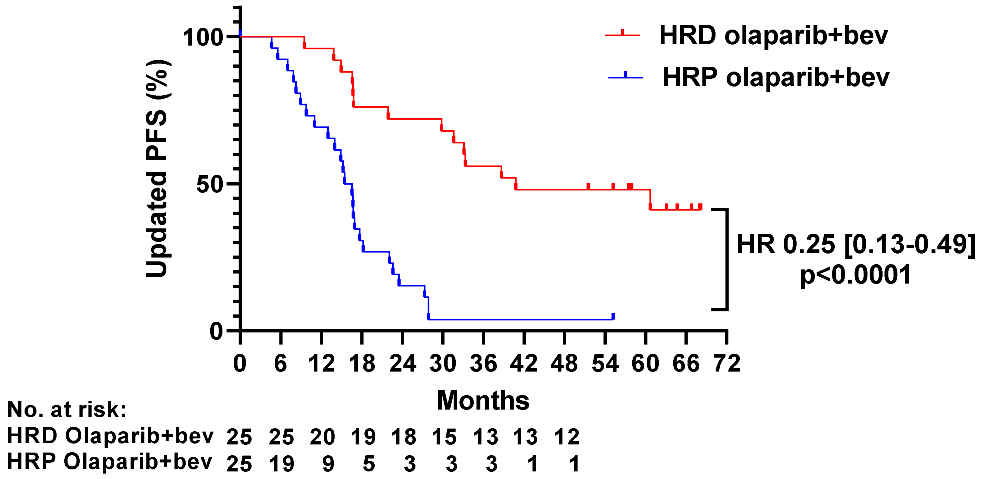

Supplementary Figure 2. Kaplan-Meier estimates of PFS for PAOLA-1 patients with tumor called borderline by the shallowHRD according to homologous recombination status as determined with shallowHRDv2 in the olaparib+bevacizumab treatment arm.

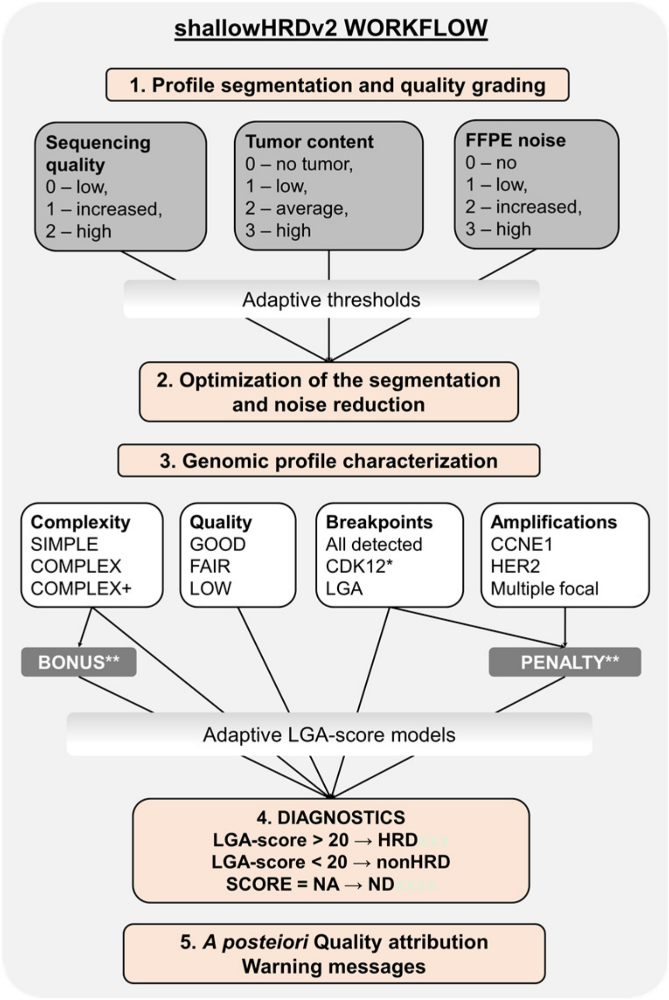

Supplementary Figure 3. Conceptual workflow of shallowHRDv2 pipeline.

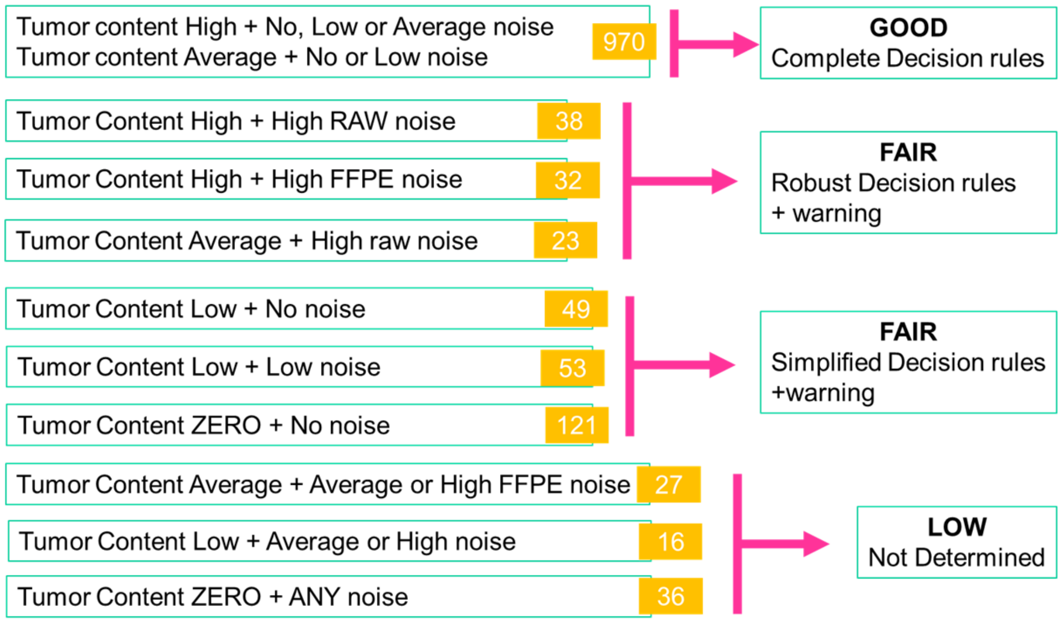

Supplementary Figure 4. Training set and quality attribution used for modulation of the decision rules. The numbers refer to the number of cases in the training set with corresponding tumor content and noise characteristics.

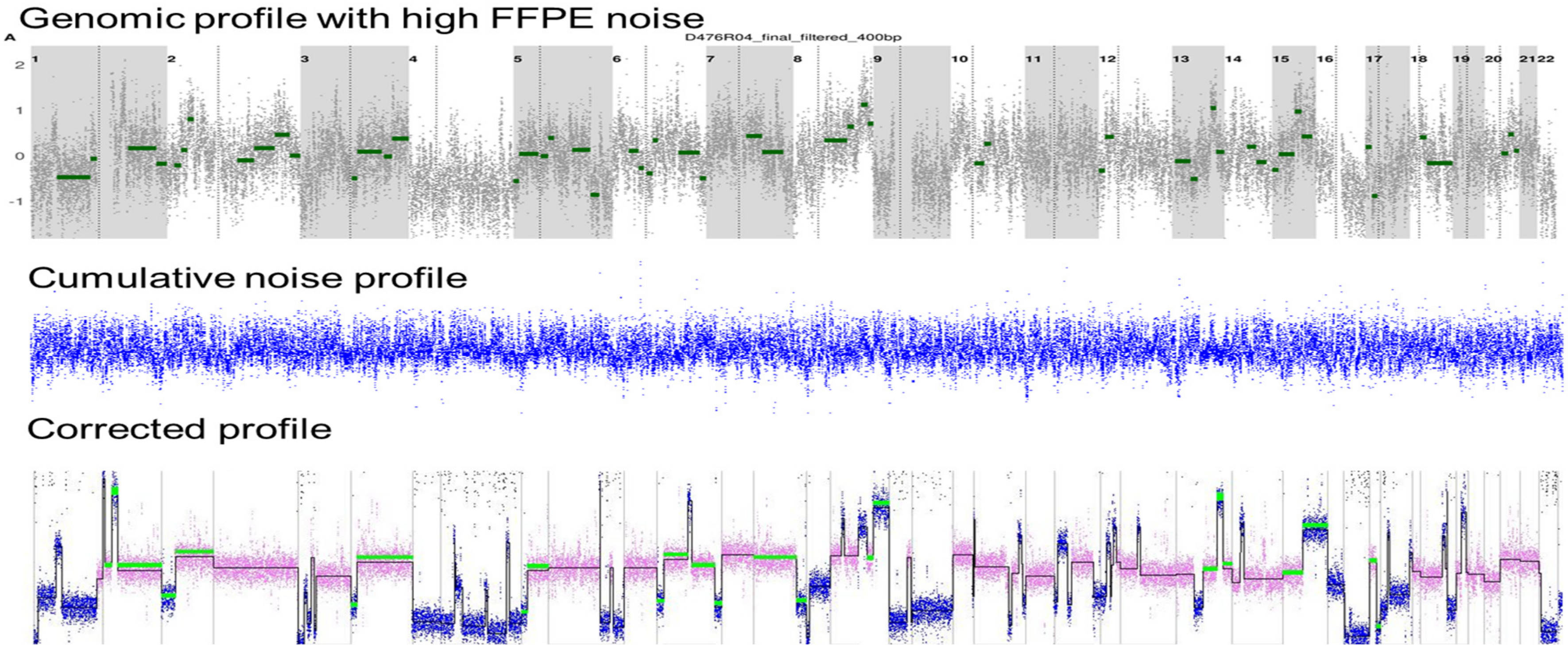

Supplementary Figure 5. Example of sWGS CNA profile with high FFPE noise, cumulative FFPE noise profile and the profile from the upper panel after correction.

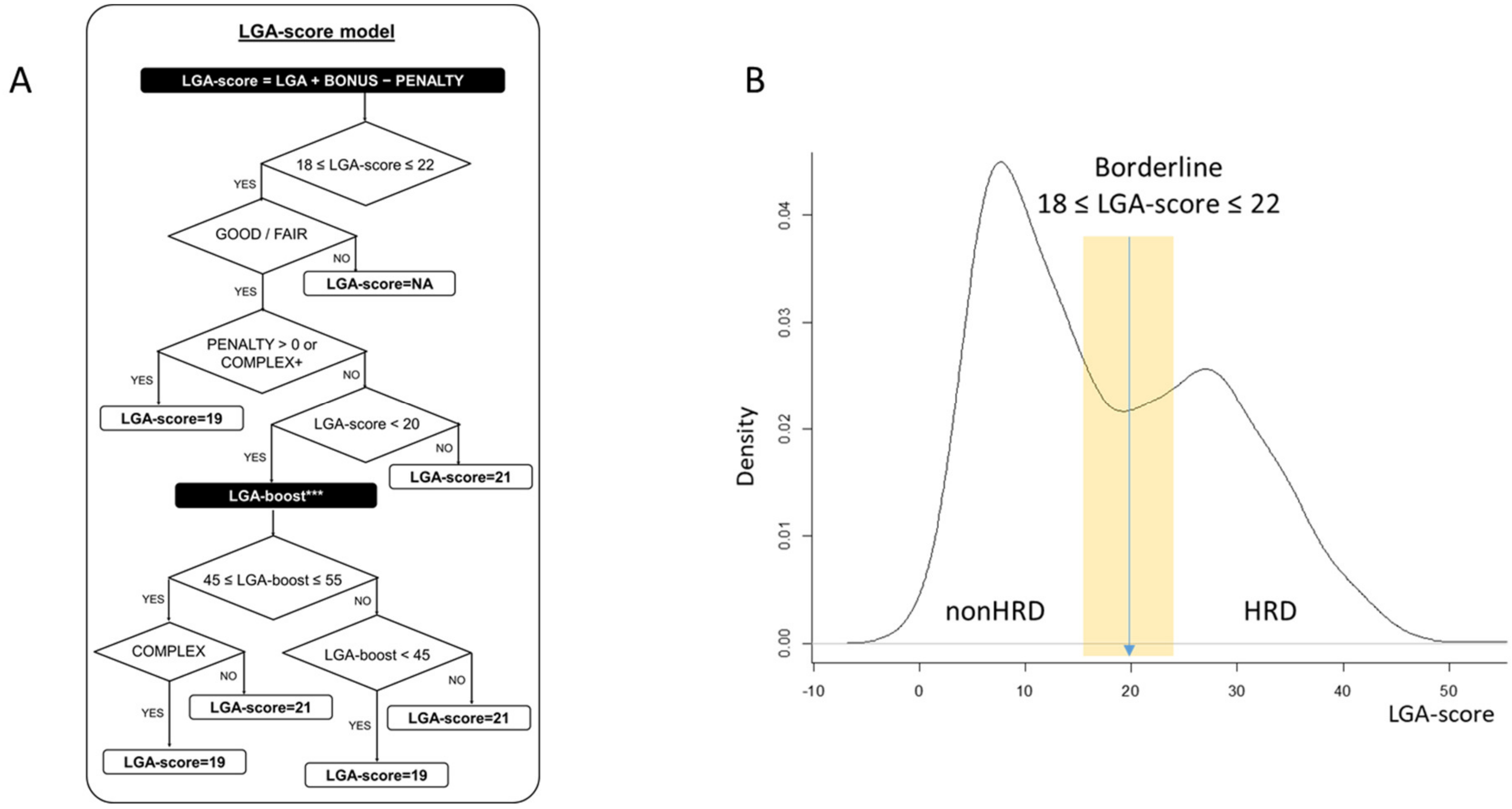

Supplementary Figure 6. Adaptive model of LGA-score evaluation (A) and LGA-score distribution in the training data set (B).

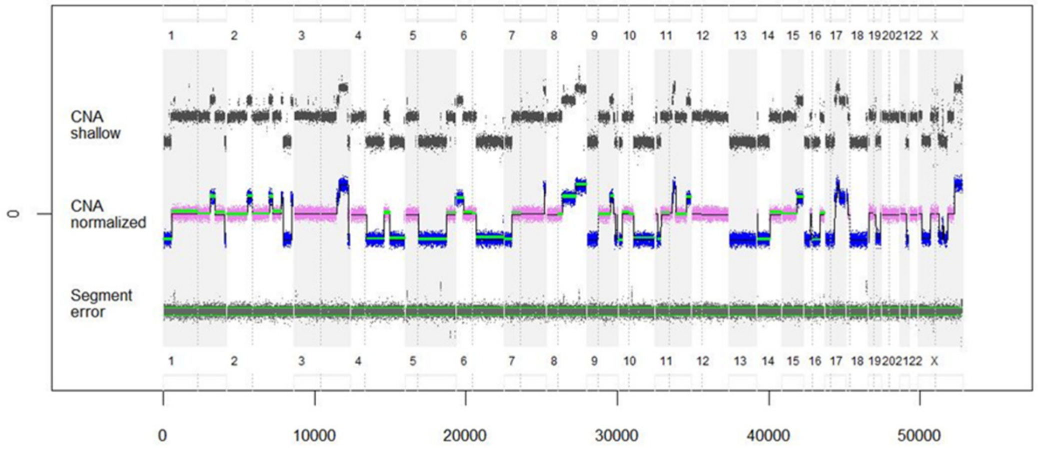

Summary Homologous Recombination Deficiency

HRD status =	HRD	Error rate <5% in 1000 samples
LGA-score =	27	SCORE>20 -> HRD / SCORE<20 -> nonHRD
CCNE1/HER2	Not altered	+++ amplified; +/++ gain. If +++ nonHRD is likely
shallowWGS coverage	NA	< 0.5 Low coverage / > 1 Optimal coverage
Tumor content	High	High / Average / Low / No tumor
Noise level	Low	Low / Moderate / High / Extreme high
LGA number	22 +/- 0	

WARNING: COVERAGE UNKNOWN

Supplementary Figure 7. Report example for shallowHRDv2.
